# A Comparison of Quality and Reliability of YouTube Videos About the MRI Technique and Its Various Aspects

**DOI:** 10.7759/cureus.63769

**Published:** 2024-07-03

**Authors:** Keyur Ranpariya, Sheethal Seelamanthula, Julia A Duca, Taseal R Ahmed, Pravinkumar Bharde

**Affiliations:** 1 Pediatrics and Neonatology, Uttambhai Nathalal Mehta Children Hospital, Surat, IND; 2 Graduate Medical Education, Sri Padmavathi Medical College for Women, Sri Venkateshwara Institute of Medical Sciences (SVIMS), Tirupati, IND; 3 Neurology, St. George's University School of Medicine, St. Georges, GRD; 4 Radiology, Tesla Diagnostics, Hyderabad, IND

**Keywords:** video power index, global quality score, youtube, machine phobia, mri process phobia, mri machines

## Abstract

Introduction: The Magnetic Resonance Imaging (MRI) machine is a subset of nuclear magnetic resonance imaging technology that produces images of the body using magnetic field gradients. The MRI Machine has two components: the computer-based control centre room and the adjacent MRI machine room where the patient undergoes the scan.

Aims: This study aimed to assess the quality and reliability of YouTube videos about MRI machines, MRI scans, and MRI claustrophobia and compare the quality and reliability of the videos among different types of uploaders.

Methodology: The YouTube Search Algorithm and a Google Sheets questionnaire were used to evaluate 10 videos that satisfied the inclusion criteria of the study. The video analytics included were title, number of views, likes and dislikes, comments, duration, source, and content. The quality of each video was established using the Global Quality Score (GQS), Reliability Score, and Video Power Index (VPI), where each quantifier went through statistical analysis using SPSS software, version 21.0 (IBM Corp., Armonk, NY) to determine if there was any significance.

Results: In order to determine statistical differences between the groups, the Kruskal-Wallis test was used on the quantifiers GQS, reliability score, and VPI to generate p-values. The p-value for VPI is 0.467, GQS is 0.277, and reliability is 0.316. All the p-values are greater than 0.05, showing that there is no statistical support for any significant difference between the groups in their VPI, GQS and reliability scores.

Conclusions: YouTube videos with high-quality and reliable information on MRI machines, MRI procedures, and claustrophobia, especially those uploaded by clinicians and hospitals, can provide correct information, helping patients decide to undergo these procedures and alleviate claustrophobia.

## Introduction

Magnetic resonance imaging (MRI) is a non-invasive radiology investigation and painless scanning technique with high spatial resolution; it uses strong magnetic fields, radio waves, and non-ionizing radiation, and generates detailed images of the targeted organs. It is considered better and safer than conventional X-rays, as it is specifically sensitive to soft tissue structures in the body [1].

The application of MRI has played a significant role in enhancing radiological diagnoses and facilitating improved medical care. However, in some patients, it has been associated with claustrophobia, the fear of closed or confined places; this affects 7.7-12.5% of the population [2]. The MRI process requires a patient to lie still on a table, inside a large magnetic tube for about 15-90 minutes. Hence, patients may develop symptoms of claustrophobia, such as anxiety, sweating, difficulty breathing, trembling, dry mouth, and chest pain, leading to a failed scan. Treatment options for claustrophobia include cognitive behavioral therapy and medicine. Open MRI scanning machines can be used as an alternative for patients with claustrophobia. 

The Internet enables access to a wide range of sources of information. When it comes to healthcare, patients and their relatives utilize these sources to learn more about diseases and laboratory investigations. Videos have the advantage of better explanation compared to articles because of their images and animations. YouTube is one such video-sharing platform that offers free access to millions of videos, and it exceeds two billion views per day. Physicians, institutions, patients, and influencers use it as a platform for sharing their knowledge, personal experiences, and treatments [3].

While some might consider this a benefit, it also raises concern. The information shared in videos can be incomplete or unreliable yet influence a patient’s decision-making regarding their treatment and medical care. This peer-reviewed article focuses on YouTube videos about MRI scanning and assesses the quality of their information using global quality scores (GQS) and reliability scores (DISCERN).

## Materials and methods

This was a cross-sectional observational study that was conducted in April 2023. Keywords such as “MRI machine,” “MRI scan,” “MRI scan video,” “MRI scan fear,” “MRI machine phobia,” and “MRI examination” were used to search for videos. A questionnaire was prepared using Google Forms, and the data was extracted into Google Sheets. A statistical analysis was then performed using SPSS software, version 21.0 (IBM Corp., Armonk, NY). Any video that did not meet all of the inclusion criteria was excluded after the preliminary selection. Only videos that met the criteria were evaluated. They were further assessed for their title, number of views, likes, dislikes, and comments, and their duration, source, and content. The video inclusion criteria were: (1) Relevance to the topic (videos having information about MRI machines or scans); 2. Discussion of MRI indications, procedures, MRI-related claustrophobia, claustrophobia-related treatment, precautions, and alternative investigations; 3. Use of English or Hindi. Furthermore, the content was analyzed using the GQS, VPI, and DISCERN [4]. A p-value was calculated for all ratings.

## Results

A total of 70 videos were evaluated, 64 of which were assessed for various factors after the application of inclusion and exclusion criteria and the removal of repetitions. Table 1 highlights the characteristics of the YouTube videos that were analyzed. The number of views, likes, dislikes, and comments were calculated to determine how popular the videos were, along with the time since they were uploaded. Of the total videos, 52 (92.2%) had been uploaded more than a year ago.

**Table 1:** Characteristics of YouTube videos analyzed

Time since uploaded	
More than a week to one year (7-365 days old)	5 (7.8%)
More than one year (> 365 days)	59 (92.2%)
Popularity	
Total no. of views	50622529
Total no. of likes	276539
Total no. of dislikes	15960
Total no. of comments	10562

Table 2 highlights the type of uploader. In this analysis, 29 (45.3%) videos were uploaded by a hospital, which made up the largest percentage. Healthcare organizations, patients, and manufacturing companies uploaded three (4.7%) videos, making up the lowest percentage.

**Table 2:** Type of uploader

Doctor	5 (7.8%)
Hospital	29 (45.3%)
Healthcare organization	3 (4.7%)
Patient	3 (4.7%)
Manufacturing company	3 (4.7%)
Other	21 (32.8%

Table 3 highlights the MRI scan procedural parameters and information that were covered in the videos. Of the total videos, 50 (78.13%) conveyed information regarding the MRI scan. However, none of the videos addressed mortality relating to the procedure or the claustrophobia that patients may experience during the scan. Additionally, 29 (45.31%) videos contained information regarding precautions.

**Table 3:** Information being “discussed” by the YouTube videos

Description of MRI machine and its parts	26 (40.63%)
Description of indications of MRI scan	14 (21.88%)
Description of MRI scan procedure	50 (78.13%)
Description of precautions before the MRI procedure	29 (45.31%)
Description of precautions/care after the MRI procedure	7 (10.94%)
Description of contraindications of MRI scan	12 (18.75%)
Description of complications/adverse events/accidents?	3 (4.69%)
Information about mortality	0 (0%)
Information about people/patient's sharing their own experience	17 (26.56%)
Does it address the issue to claustrophobia?	22 (34.38%)
Does it describe how to prevent (preparation before the scan) claustrophobia?	19 (29.69%)
Does it describe treatment (after the scan) of claustrophobia caused during MRI scan?	0 (0%)
Does it describe alternate investigation since patient has claustrophobia caused during MRI scan?	5 (7.81%)
The post has a promotional content by pharmaceutical/machine company or by doctors?	3 (4.69%)

Table 4 compares the GQS, DISCERN, and VPI based on the type of uploader. Videos uploaded by hospitals were generally of good quality and flow and were useful for patients; they had a median GQS of 4 (2.5, 4.5) but were of moderate reliability with a median DISCERN of 3 (2, 4). However, videos uploaded by doctors were of limited use for patients, with a median GQS of 2 (2, 4.5) and poor reliability with a median DISCERN of 2 (2, 3.5). The p-values for VPI (0.467), GQS (0.277), and DISCERN (0.316) show that there is no significant difference between the groups.

**Table 4:** Comparison of GQS, reliability score and VPI based on type of uploader GQS: Global quality score; VPI: Video power index

	Doctors (n=5)	Hospital (n=29)	Healthcare organization (n=3)	Patient (n=3)	Manufacturing company (n=3)	Other (n=21)	p-value & test used
	Median (IQ1, IQ3)	Median (IQ1, IQ3)	Median (IQ1, IQ3)	Median (IQ1, IQ3)	Median (IQ1, IQ3)	Median (IQ1, IQ3)	Test Used: Kruskal-Wallis Test
VPI	85.18 (1.445,4940.955)	101.62 (24.865,242.725)	200.87 (109.73,.)	21.78 (12.39,.)	2546.75 (42.93,.)	126.35 (36.85,771.27)	p-value = 0.467
GQS	2 (2,4.5)	4 (2.5,4.5)	3 (1,.)	3 (2,.)	3 (3,.)	3 (2,3)	p-value = 0.277
Reliability Score	2 (2,3.5)	3 (2,4)	2 (1,.)	2 (1,.)	3 (2,.)	3 (2,3)	p-value = 0.316

## Discussion

This study analyzed the quality of YouTube content about MRI machines and their associations, specifically with claustrophobia. A total of 70 videos were evaluated; only 64 met the inclusion criteria, similar to a study evaluating YouTube video content quality for medical education purposes by Mutlu and Arik that used 65 videos [5]. In other studies, Batar et al. used 52 videos, Crutchfield et al. used 142 videos, Di Bello et al. 156 videos, and Desai et al. 92 videos that met inclusion criteria [6-9].

This study found that the majority of the uploaded videos 92.2% (n=59) were more than one year old compared to the remaining videos that were more than one week to one year old 7.8% (n=5). In similar studies, there was less emphasis on the publishing time of the video and more focus on content quality [5-9]. Since most of the videos were over a year old, this might imply the potential for physicians, hospitals, and health organizations to use YouTube more efficiently to provide timely information. This could be especially useful when trying to explain the concerns of those suffering from MRI-induced claustrophobia, as potential patients may be unaware of new advances in MRI technologies, such as open MRIs and upright MRIs, or approaches such as meditation that may improve their experience.

The popularity of the videos showed their widespread reach, with a total number of 50,622,529 views; 276,539 likes and 15,960 dislikes; and 10,562 comments. The survey found that 45.3% (n=29) videos were uploaded by hospitals, 32.8% (n=21) videos were uploaded by other channels, and 7.8% (n=5) videos were uploaded by doctors. The remaining 4.7% (n=3) videos were uploaded by a healthcare organization, a patient, and a manufacturing company. In contrast, the uploader distribution in a study by Mutlu and Arik was doctors 11% (n=7), MRI technologists 7% (n=5), hospitals 8% (n=5), health channels 28% (n= 5), patients 28% (n=18), and others 18% (n= 12) [5]. Another study by Desai et al. categorized videos into physician-led videos 35% (n=35), other medical professionals 20% (n=20), non-medical practitioners 13% (n=13), surgical technique 16% (n=16), patient testimonials 9%(n=9), and news 7%(n=7) [9]. In a study by Batar et al. regarding hallux valgus, the main uploaders were medical companies 28.8% (n=15), non-surgical physicians 38.5% (n=20), and surgeons 30.8% (n=16) [6]. Crutchfield et al. found in their study on femeroacteabular impingement that the videos were educationally oriented 48.6% (n=69), physician-sponsored 30.3% (n=43), testimonials 14.8% (n=21), techniques 4.2% (n=6), and news 2.1% (n=3) [7].

Moreover, the YouTube videos provided information on the MRI procedure. The study found that 78.13% (n=50) provided descriptions of MRI scan procedure, 45.31% (n=29) descriptions of precautions before MRI procedure, and 40.63% (n=26) videos described MRI machines and their parts. While none of the videos provided information on mortality or treatments for claustrophobia, only three (4.69%) videos were promotional content by a pharmaceutical or machine company or by doctors. While Mutlu and Arik’s study found that 30.77% of the content distribution was on MRI-associated claustrophobia, 26.25% on sharing experiences, and 43.08% on overcoming fear, Desai et al., in their study on YouTube quality regarding ulnar collateral injury, found that only 3% of the analyzed videos discussed indications for ulnar collateral ligament repair, with 4% mentioning the need to avoid postoperative stress [5,9]. Similarly, the study by Crutchfield et al. found that there was little mention of postoperative restrictions, rehabilitation protocols, or surgical complications in the analyzed videos [7]. These findings that key information is lacking from the majority of informational videos indicate a knowledge gap for patients who use YouTube as a resource for medical information, as well as a need for more reliable and comprehensive video content about medical procedures and pathologies.

The VPI, GQS, and reliability scores did not show any significant differences among the types of uploaders when comparing doctors, hospitals, healthcare organizations, patients, manufacturing companies, and others. This differed from a similar study by Crutchfield et al., where there was a correlation between video duration length and the quality of the video content [7]. This was not possible in this study due to the limited sample size. In similar studies on MRI and claustrophobia, Batar et al. and Mutlu and Arik found that the DISCERN score and GQS were both significantly higher in professional videos [5,6]. In this study, the uploaders were analyzed separately, while Mutlu and Arik divided the uploaders into professionals (hospitals, doctors, and MRI technicians) and non-professionals for GQS and DISCERN comparisons [5].

Limitations

Only 64 videos were reviewed in total. One of the inclusion conditions was that the language be English and Hindi and that the videos be of limited duration. As a result, videos with comparably more accurate information may have been excluded. Further, because this study was conducted in a single day, it was confined to a snapshot of demographic data of videos on that specific day. Over time, the total number of likes, comments, and views may fluctuate, and more videos with higher-quality material relevant to the chosen keywords may be added to YouTube. The GQS for the same video may change among observers.

## Conclusions

This study found that there is no significant difference in the information uploaded by educational organizations, healthcare professionals, patients, or other individuals on MRI. Because of its large audience, YouTube should be utilized as a platform for healthcare professionals to share and educate people with credible information. Hence, there is a need for physicians and healthcare organizations to submit verified and high-quality educational videos on YouTube with clear and comprehensible language. Information about claustrophobia and its management should also be explored. These videos should also emphasize two other aspects: individuals should not self-diagnose, and should seek clinics, hospitals, and physicians for treatment.
